# Effect of trust in primary care physicians on patient satisfaction: a cross-sectional study among patients with hypertension in rural China

**DOI:** 10.1186/s12875-020-01268-w

**Published:** 2020-09-21

**Authors:** Wenqin Chen, Yingchao Feng, Jiyuan Fang, Jin Wu, Xianhong Huang, Xiaohe Wang, Jian Wu, Meng Zhang

**Affiliations:** 1 School of Medicine, Hangzhou Normal University, No. 2318, Yu-hang-tang Road, Hangzhou, China; 2 College of Public Health, Zhengzhou University, Zhengzhou, China

**Keywords:** Patient trust, Patient satisfaction, Hypertension management, Rural area

## Abstract

**Background:**

In rural areas of China, hypertension is on the rise and it is drawing the Chinese government’s attention. The health outcomes of hypertension management can be positively impacted by patient satisfaction with primary care physicians (PCPs), and the influence of patient trust on satisfaction cannot be ignored. This study aimed to analyze the effect of trust in PCPs on patient satisfaction among patients with hypertension in rural China, and the influence of patients’ socio-demographic characteristics and hypertension-management-related factors.

**Methods:**

A multi-stage stratified random sampling method was adopted to investigate 2665 patients with hypertension in rural China. Patient trust and satisfaction were measured using the Chinese version of the Wake Forest Physician Trust Scale and the European Task Force on Patient Evaluation of General Practice. Multiple linear regression was used to analyze the factors influencing patient satisfaction, and structural equation modeling was conducted to clarify the relationships among patient trust and patient satisfaction with PCPs.

**Results:**

Patients’ trust in their PCPs’ benevolence had a positive main effect on all three satisfaction dimensions (clinical behavior: β = 0.940, *p* <  0.01; continuity and cooperation: β = 0.910, *p* <  0.01; and organization of care: β = 0.879, *p* <  0.01). Patients’ trust in their PCPs’ technical competence had a small negative effect on all three satisfaction dimensions (clinical behavior: β = − 0.077, *p* <  0.01; continuity and cooperation: β = − 0.136, *p* <  0.01; and organization of care: β = − 0.064, *p* <  0.01). Patient satisfaction was also associated with region, gender, insurance status, distance from the nearest medical/health-service institution, and number of visits to PCPs in the past year.

**Conclusions:**

Patients focused more on physicians’ benevolence than on their technical competence. Hence, medical humanities and communication skills education should be emphasized for PCPs. Regarding region-based and health-insurance-based differences, the inequities between eastern, central, and western provinces, as well as between urban and rural areas, must also be addressed.

## Background

Hypertension is a common chronic disease and is the most important risk factor for cardiovascular and kidney disease [1]. Worldwide, over one in five adults have high blood pressure (of whom 52% have uncontrolled blood pressure), and there are 9.4 million deaths from hypertension complications annually [2]. In 2018, there were over 200 million people with hypertension in China, and this figure is rising at a rate of 10 million people per year [3]. The number of adults from rural areas in China presenting with hypertension has increased rapidly: the prevalence rate was 18.94% from 2004 to 2006, 21.24% from 2007 to 2009, and 26.68% from 2010 to 2013 [4].

Since 2009, China has considered the health management of patients with hypertension to be a “national basic public health services project”; a basic public health service for key populations (e.g., older adults, women, and children), focusing on key diseases (e.g., chronic or infectious diseases) and meeting residents’ basic health needs. The National Basic Public Health Service Project Regulations (2011) stipulate that primary-level medical and health institutions should provide community health management for hypertension patients. The rural health-service system services 800 million Chinese rural dwellers and directly affects the health status and service utilization of the rural population [5]. The health administration’s “Guidelines for the Management of Hypertension in China” also propose emphasizing hypertension prevention and management in rural areas [6]. Long-term adherence to lifestyle improvement is the cornerstone of associated treatment approaches, and rational use of antihypertensive drugs is key to achieving normal blood pressure [6]. Nonadherence to treatment can be due to the patient’s lack of knowledge about the provider’s decision-making process and low physician credibility [7]. To ensure the effectiveness of this treatment, establishing an enduring and harmonious relationship marked by mutual understanding between doctors and patients is essential [8]. Indeed, trust is the foundation of the doctor-patient relationship, and given that patient satisfaction is an indicator of health service quality, high levels of trust and patient satisfaction indicate a good relationship between patients and service providers [9]. However, studies have shown that primary-level institutions in the rural health-service system – in which township health centers or community health-service centers represent primary hubs, and village clinics or community health-service stations represent the lowest level – have weak service capabilities and low resident satisfaction [10]. Importantly, this is not conducive to effective long-term follow-up management of rural patients with hypertension and has a negative impact on the prevention and treatment of hypertension.

Patient satisfaction refers to people’s assessment of the health-care services that they receive, and is based on their requirements regarding health, disease, quality of life, etc. [11]. As a patient-reported outcome and a major component of health-care quality, patient satisfaction can impact therapeutic outcomes [12]. For patients with hypertension, treatment satisfaction may provide insight into attitudes toward hypertension treatment [13]. Such satisfaction is associated with higher adherence to antihypertensive drugs and improved health-related quality of life [2].

Several studies have investigated the factors that influence patient satisfaction regarding primary health-care services and have shown that regular visits to a particular general practitioner (GP), distance from a primary health-care center, age, gender, socioeconomic status, and health status are associated with patient satisfaction [14, 15]. Along with these objective factors concerning patients’ sociodemographic characteristics and health status, patient satisfaction is also heavily influenced by patients’ subjective perceptions and interpersonal relationships [16]. Notably, patient trust is the foundation of the doctor-patient relationship and leads to perceiving doctors as reliable, acting in the patient’s best interests, and providing support and assistance regarding the patient’s health problems [17]. Patient trust has been shown to be an important factor in fostering satisfaction [2, 18–20].

However, existing China-based research regarding the factors affecting patient satisfaction has mostly focused on urban areas, with little attention to physicians in rural primary health-care institutions. The few studies in this latter category have been limited to specific geographic areas [21–24]. Although researchers largely acknowledge that trust impacts satisfaction, some studies have only performed this analysis from a qualitative viewpoint, while others have used unsuitable or limited questionnaires [25–28]. Univariate and regression analyses have been commonly used for analysis [2, 29–32]. Given that social science research cannot directly measure trust and satisfaction, measurement error is inevitable [33]. To address this, recent studies have applied structural equation modeling (SEM) when evaluating patient trust and satisfaction [18, 34]. However, few studies have analyzed the relationship between trust and satisfaction from the perspective of refining their internal dimensions. Thus, in our study, different dimensions of the two variables were scientifically divided according to literature research based on the mature scales, and the influence among them was explored by using the structural equation model.

In the present study, the Chinese version of the Wake Forest Trust Scale (WFPTS-C) was used to measure trust among rural-based patients with hypertension. This scale was developed by Hall and has been used in several countries to examine trust in primary care providers, including physicians, comprised of 4 dimensions: fidelity, competence, honesty and global [29, 30]. The Chinese version has previously been shown to have beneficial psychological attributes among patients by Dong and Bao [35]. And a two-dimensional model (comprising “benevolence” and “technical competence”) has been verified as a better fit to the data among Chinese patients than Hall’s four-dimensional model or Bachinger’s one-dimensional model [17, 36, 37].

To measure satisfaction, we used the European Satisfaction Survey Scale (EUROPEP), which is a comprehensive tool representative of international standards that measures service satisfaction and was developed through a rigorous design process [38]. The EUROPEP does not evaluate a specific visit or doctor, but rather, patients’ satisfaction with doctors regarding services provided “over the last 12 months” [30]. As this scale measures continuity-related aspects (i.e., repeated visits over 12 months), it captures patients’ satisfaction with the normative management requirements for hypertension, meaning it can be applied to primary-health-care institutions. Generally, EUROPEP scores reflect two dimensions: clinical behavior (items 1 to 16) and organizational mechanisms (items 17 to 23) [39, 40]. Of these, the former items can be divided into “relation and communication,” “medical care,” and “information and support,” and the latter items into “continuity and cooperation” and “organization of care” [41].

**Fig. 1:**
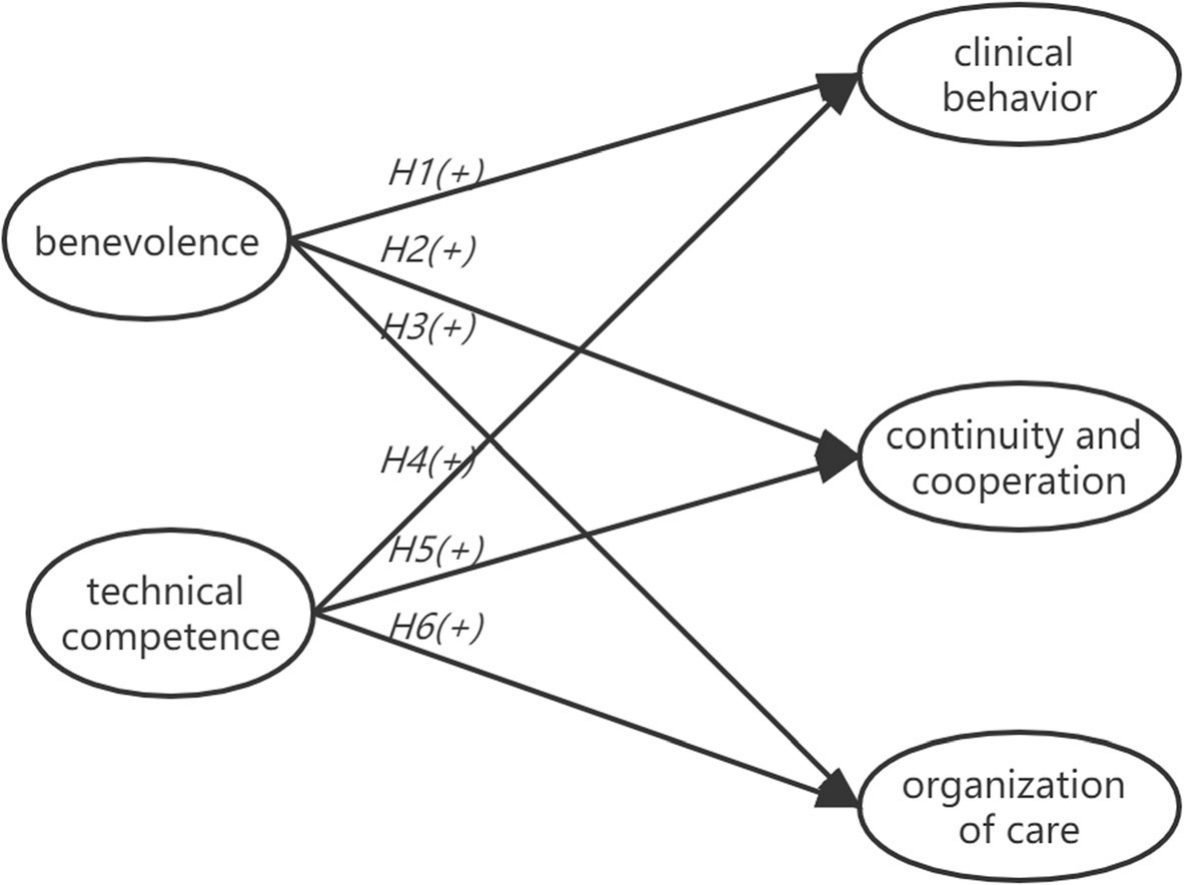
Structural framework of the theoretical relationships

We hypothesize that among rural-based patients with hypertension, trust in primary care physicians (PCPs) will have a positive impact on satisfaction. For the internal dimensions, we hypothesize that trust in PCPs’ benevolence will have positive impacts on all three dimensions of patient satisfaction (clinical behavior, continuity and cooperation, organization of care; H1-H3), and patients’ trust in PCPs’ technical competence will also have positive effects (H4-H6). The framework for our theoretical relationships is shown in Fig. 1. Furthermore, we explore the influence of patients’ socio-demographic characteristics and hypertension-management-related factors on satisfaction, aiming to identify the means to promote patient satisfaction and to improve the doctor-patient relationship and the rate of hypertension control in rural areas.

## Methods

### Study design and population

This study comprised a cross-sectional analysis of rural Chinese hypertension patients and analyzed the effect that trust in PCPs has on patient satisfaction. Using a multistage stratified random sampling method, between February 2017 and May 2018 we surveyed 2665 hypertension patients (response rate: 99.6%; 2665/2675) receiving care from rural primary-health-service institutions. Any questionnaire with a completion rate of 90% was regarded as efficient. The scores of efficient questionnaires were accounted, and missing data were replaced with the median. We selected three Chinese provinces to obtain representative samples from the eastern, central, and western regions: Zhejiang, Henan, and Shaanxi. Next, the counties of each province were divided into two categories (high and low) based on the level of economic development, and one sample county was randomly chosen from each category. Then, three townships from each county were randomly selected as sample townships by classifying each township as economically developed, moderately developed, or less developed and choosing one from each category. Next, three sample villages were chosen based on their distance from township hospitals (far, medium, and close). Finally, in every village, using the relevant primary health service institution’s hypertension management archives, a random sample of 50 hypertension patients was selected for the survey. Our sample size met the requirement that sampling (using SEM) should contain at least 20 observations per variable of analysis according to the heuristics, and that the number of samples for each unit should be more than 30 (that is, a large sample size) [42]. Additionally, we increased the sample size by approximately 10% to account for unforeseeable factors.

Hangzhou Normal University’s scientific research ethics committee reviewed and approved the study protocol. Prior to the administration of the questionnaires, oral informed consent was first obtained from all patients considering the age and the education level of patients with hypertension in rural areas. All participants satisfied the following inclusion criteria: (a) had received hypertension management for more than 1 year; (b) had a normal intelligence quotient; (c) did not have any brain trauma or brain disease, visual or auditory dysfunction, or psychiatric disorder; and (d) could speak or read Chinese.

### Measures

The questionnaire was distributed by trained interviewers. All subjects were asked a core set of questions regarding their socio-demographic characteristics (i.e., age, gender, household register, marital status, level of education, per-capita annual household income, and health insurance), hypertension management (i.e., number of visits to PCPs in the past year, blood pressure, and distance from the nearest health-service institution), and self-reported health status. Patients’ trust and satisfaction with their PCPs were measured using the WFPTS-C and the EUROPEP, respectively.

### Chinese version of the wake Forest physician trust scale

Hall et al. [43, 44] verified the reliability and validity of the WFPTS through a large number of empirical studies. A modified Chinese version was developed by Dong and Bao [37], comprising 10 items that are scored using a 5-point Likert scale, ranging from 1 (“strongly disagree”) to 5 (“strongly agree”; scoring is reversed for items 2, 3, 7, and 8). The Chinese scale’s two-dimensional structure, “benevolence” and “technical competence,” has been verified in previous research. The overall score is computed through an unweighted summation of the individual item scores, with higher scores reflecting greater trust. In the present study, the total score for the trust scale ranged from 10 to 50, and the median (interquartile range) was 24 (4).

### European task force on patient evaluation of general practice

The EUROPEP is a comprehensive 23-item questionnaire that measures patients’ satisfaction with their general medical services [40] and assesses patients’ opinions of their regular GP, based on their experiences over the previous 12 months. Items are scored using a 5-point Likert scale (1 = “poor,” 3 = “acceptable,” 5 = “excellent”). The EUROPEP has been tested in 16 European countries, and its technical quality has been verified [41]. A revised Chinese version was created by Han [45]. We divided the scale into three dimensions: clinical behavior (15 items), continuity and cooperation (3 items), and organization of care (5 items concerning facilities, availability, and accessibility). In the present study, the total score for the satisfaction scale ranged from 23 to 96, and the median (interquartile range) was 50 (21).

### Reliability and validity of the Chinese version of the wake Forest physician trust scale and the European task force on patient evaluation of general practice

Responses to all 10 and 23 items of the C-WFPTS and EUROPEP, respectively, were entered into an exploratory factor analysis model. The principal component extraction method was used to extract the components of each scale. Consequently, the two-component model of C-WFPTS and the three-component model of EUROPEP were determined to explain 53.75 and 63.601% of the total variance, respectively. The components were consistent with previous researches using the two scales. The two components of C-WFPTS were “Benevolence” and “Technical competence”, and the three components of EUROPEP were “Clinical behavior” , “Continuity and cooperation” and “Organization of care”. Factor loadings for the two scales were shown in Tables 1 and 2. The Kaiser-Meyer-Olkin (KMO) test for sampling adequacy returned values of 0.833 and 0.973, and the Bartlett’s test of sphericity χ^2^ returned 7135.817 and 42,086.746 (*p* <  0.001), indicating that the scales contained good construct validity. Both scales and their dimensions also showed favorable internal consistency, ranging from 0.728 to 0.958.

**Table 1:** Factor analysis with factor loadings for C-WFPTS

	Component	
	1	2
1. My doctor will do whatever it takes to provide me all the care I need.	0.72	
2. Sometimes my doctor cares more about what is convenient for him/her than about my medical needs.	0.80	
4. My doctor is extremely thorough and careful.	0.70	
6. My doctor is totally honest in telling me about all of the different treatment options available for my condition.	0.73	
8. My doctor only thinks about what is best for me.	0.66	
3. My doctor’s medical skills are not as good as they should be.		0.74
5. I completely trust my doctor’s decisions about which medical treatments are best for me.		0.51
7. Sometimes my doctor does not pay full attention to what I am trying to tell him/her.		0.76
9. I have no worries about putting my life in my doctor’s hands		0.75
10. All in all, I have complete trust in my doctor.		0.56
Variation %	35.01	18.74

Kaiser-Meyer-Olkin measure of sampling adequacy: 0.833

Bartlett’s test of sphericity: *χ*^*2*^: = 7135.817, *p* <  0.001

**Table 2:** Factor analysis, with factor loadings, for EUROPEP

	Component		
	1	2	3
1. Making you feel you had time during consultations	0.68		
2. Interest in your personal situation	0.77		
3. Making it easy for you to tell him or her about your problems	0.75		
4. Involving you in decisions about your medical care	0.63		
5. Listening to you	0.77		
6. Keeping your records and data confidential	0.67		
7. Quick relief of your symptoms	0.71		
8. Helping you to feel well so that you can perform your normal daily activities	0.74		
9. Thoroughness	0.73		
10. Physical examination	0.73		
11. Offering you services for preventing diseases	0.64		
12. Explaining the purpose of tests and treatments	0.68		
13. Telling you what you wanted to know about your symptoms and/or illness	0.71		
14. Help in dealing with emotional problems related to your health status	0.62		
15. Helping you understand the importance of following his or her advice	0.64		
16. Knowing what s/he had done or told you during previous contacts		0.83	
17. Preparing you for what to expect from a specialist or hospital care		0.80	
18. The helpfulness of staff (other than the doctor)		0.51	
19. Getting an appointment to suit you			0.63
20. Getting through to the practice on the phone			0.60
21. Introducing you to other doctors in time or arranging a referral to the best hospital, if necessary			0.76
22. Waiting time in the waiting room			0.59
23. Providing quick services for urgent health problems			0.78
Variation %	53.23	5.74	4.64

The Kaiser-Meyer-Olkin measure of sampling adequacy: 0.973

Bartlett’s test of sphericity: *χ*^*2*^: = 42,086.746, p <  0.001

### Statistical analysis

In the initial analysis, outlier data and multicollinearity were assessed before proceeding. The existence of outliers was identified using Cook’s distance [46]: if the observed Cook’s distance was greater than 0.5, the point was considered an outlier or strong influence point. Our analysis returned a maximum Cook’s distance of 0.036, indicating no outlier data. Next, multicollinearity was tested by considering tolerance rate and the variance inflation factor (VIF) [47]. The results showed no tolerance rate below 0.10 or VIF above 10; all tolerance values were above 0.77 and all VIFs were below 1.30, indicating no multicollinearity.

We used Cronbach’s α values to test the reliability of the scale and factor analysis to test structural validity. Categorical variables were presented through frequencies and percentages. The normality of the distribution of the continuous variables was tested using a one-sample Kolmogorov-Smirnov test. Continuous variables with normal distribution were presented as means ± standard deviations; non-normal variables were reported as medians (interquartile range). Comparisons of continuous variables (scores for patient trust and satisfaction) were conducted using *t*-tests and one-way analyses of variance (ANOVA) tests, while Wilcoxon rank-sum tests (the Mann-Whitney *U* test and Kruskal-Wallis test) were used for non-normally distributed values. *p* values of < 0.05 indicated statistical significance. Multiple linear regression analysis was conducted, with patient satisfaction as the dependent variable and patient trust, as well as its two dimensions, as the independent variable. Model covariates were selected from those that returned a *p*-value of less than 0.2 in the univariate analysis. Next, SEM was conducted to test our hypotheses. SEM can be used to measure latent variables, and it allows the measurable variable and the latent variables to be placed in a common model, which can include multiple dependent variables in one measurement, reducing the error of multiple linear regression analysis. We also used several fit indices, including chi-square ratio (< 3), goodness of fit index (GFI; > 0.9), adjusted goodness of fit index (AGFI; > 0.9), normal fit index (NFI; > 0.9), and root mean square error of approximation (RMSEA; < 0.05) to evaluate overall model fitness. All analyses were conducted using SPSS 16.0 and AMOS 22.0 (SEM).

## Results

### Patients’ demographic characteristics, hypertension management, and self-reported health status

This study included a total of 2665 patients. Over half (62.4%) were female; the majority were middle-aged or older adults, with only 33 patients under 45 years of age. Most respondents were married (82.5%) and had lower than senior high school education at survey time (95.8%). As we conducted our survey in rural areas, most respondents’ insurance was provided through the New Rural Cooperative Medical Scheme (NRCMS; 75.6%), a rural dwellers’ medical mutual helping system organized, guided, and supported by local government [48]. Specific data, including data for additional main characteristics, are presented in Table 3.

**Table 3:** Characteristics of the surveyed patients

Characteristic	N	%
Region		
Eastern province	893	33.5
Central province	885	33.2
Western province	887	33.2
Gender		
Male	1002	37.6
Female	1663	62.4
Age		
< 45	33	1.2
45–59	483	18.1
60–74	1533	57.5
> 75	616	23.1
Marital Status		
Married	2138	80.2
Other	527	19.8
Level of Education		
Primary or lower	2090	78.4
Junior high school	462	17.3
Senior high school or above	113	4.2
Insurance type		
Medical Insurance for Urban Employees	72	2.7
Medical Insurance for Urban Residents	191	7.2
Basic Medical Insurance for Urban and Rural Residents	636	23.9
New Rural Cooperative Medical Scheme	1741	65.3
Other	25	0.9
Per-Capita Annual Household Income		
1 (≤ 1000 yuan)	571	21.4
2 (1000–2160 yuan)	496	18.6
3 (2161–4000 yuan)	549	20.6
4 (4001–10,000 yuan)	557	20.9
5 (> 10,000 yuan)	492	18.5
Distance from the nearest medical and health service institutions		
< 1 km	2075	77.9
1–2.99 km	550	20.6
≥ 3 km	40	1.5
Course of disease		
≤ 3 years	600	22.5
4–10 years	1248	46.8
11–20 years	664	24.9
> 20 years	153	5.7
Self-reported health status		
Bad	624	23.4
Neither good nor bad	1800	67.5
Good	241	9.0
Blood pressure		
Controlled	1699	63.8
Uncontrolled	966	36.2
No. of visits in the past year		
< 4	1251	46.9
≥ 4	1414	53.1

### Univariate analysis of factors associated with patient satisfaction

Wilcoxon rank-sum tests revealed that patient satisfaction was associated with region, gender, insurance status, per-capita annual household income, distance from the nearest medical/health-service institution, and number of visits to PCPs in the past year. Residents of eastern and central zones (*p* <  0.001), males (*p* <  0.001), recipients of medical insurance for urban residents (*p* <  0.001), and those living near a medical/health-service institution (*p* <  0.001) had better self-reported health status, made fewer visits in the past year, and had significantly lower satisfaction with their PCPs (Table 4).

**Table 4:** Univariate analysis of factors associated with patient satisfaction

Characteristic	Classification	Median (Interquartile range)
Region	Eastern province	51(18)
	Central province	50(21)
	Western province	47(24)
χ^2^ (p)		28.539 (< 0.001**)
Gender	Male	51(22)
	Female	49(19)
Z(p)		−3.480 (0.001**)
Age	< 45	52(20)
	45–59	50(24)
	60–75	50(22)
	> 75	50(18)
χ^2^ (p)		7.424 (0.060)
Marital Status	Married	50(22)
	Other	49(20)
Z(p)		−0.138 (0.890)
Level of Education	Primary or below	50(20)
	Junior high school	48(24)
	Senior high school and above	50(19)
χ^2^ (p)		2.645 (0.266)
Insurance	Medical Insurance for Urban Employees	46(22)
	Medical Insurance for Urban Residents	60(16)
	Basic Medical Insurance for Urban and Rural Residents	49(18)
	New Rural Cooperative Medical Scheme	49(23)
	Other	49(24)
χ^2^ (p)		71.755 (< 0.001**)
Per-Capita Annual Household Income Quintile	1	53(24)
	2	50(23)
	3	47(23)
	4	50(18)
	5	50(18)
χ^2^ (p)		13.490 (0.009**)
Distance from the nearest health service institution	< 1 km	50(20)
	1–3 km	49(22)
	≥4 km	48(25)
χ^2^ (p)		14.553 (< 0.001**)
Course of disease	≤ 3 years	48(23)
	4–10 years	50(21)
	11–20 years	50(20)
	> 20 years	50(18)
χ^2^ (p)		7.611 (0.055)
Self-reported health status	Bad	49(20)
	Neither good nor bad	50(20)
	Good	48(24)
χ^2^ (p)		5.559 (0.062)
No. of visits in the past year	< 4	52(22)
	≥4	48(21)
Z(p)		−5.238 (< 0.001**)

* <  0.05, ** <  0.01

### Multiple linear regression analysis

The results of our multiple linear regression analysis (Table 5 shows the assignment of demographic variables), with satisfaction score as the dependent variable, trust score as the independent variable, and after controlling for other covariates, showed that trust score (β = 0.435, *p* <  0.01), receiving medical insurance for urban residents (β = 0.133, *p* <  0.01), and living in the central province (β = 0.149, *p* <  0.01) were associated with significantly higher satisfaction scores. In contrast, the number of visits in the past year (β = − 0.121, *p* <  0.01) and distance from the nearest medical/health service institution (β = − 0.074, *p* <  0.01) were associated with significantly lower scores. In addition, males had significantly higher satisfaction scores compared to females (Table 6). We conducted a second linear regression, taking the two dimensions of trust as independent variables; this analysis showed that the score for “benevolence” was associated with significantly increased satisfaction (β = 0.532, *p* <  0.01), while “technical competence” did not feature in the model (Table 6).

**Table 5:** Assignment of demographic variables

Variable	Reference group	Assignment
Region	Western zone	Central province = 1; Eastern province = 2
Age	< 45	45–59 years = 1; 60–75 years = 2; > 75 years = 3
Insurance	Medical Insurance for Urban Employees	Medical Insurance for Urban Residents = 1; Basic Medical Insurance for Urban and Rural Residents = 2; New Rural Cooperative Medical Scheme = 3; Other = 4
Distance from the nearest health service institutions	< 1 km	1–3 km = 1; ≥ 4 km = 2
Course of disease	≤ 3 years	4–10 years = 1; 10–20 years = 2; > 20 years = 3
Self-reported health status	Bad	Neither good nor bad = 1; Good = 2

**Table 6:** Results of linear regression models examining predictors of hypertensive patients’ satisfaction with PCPs

Variable	Unstandardized beta	SE	Standardized beta	*t*	*p*	Confidence interval
**Model 1 (taking total trust score as the independent variable)**						
Constant	12.749	1.803	–	7.071	< 0.001	(9.213, 16.284)
**Trust**	1.597	0.065	0.435	24.752	< 0.001	(1.470, 1.724)
**No. of visits in the past year**	− 0.100	0.014	−0.121	−7.031	< 0.001	(−0.127, − 0.072)
**Insurance**						
Medical Insurance for Urban Employees (reference group)						
Medical Insurance for Urban Residents	6.882	0.917	0.133	7.502	< 0.001	(5.083, 8.681)
Basic Medical Insurance for Urban and Rural Residents	1.243	0.593	0.040	2.096	0.036	(0.080, 2.406)
**Region**						
Western province						
Central province	4.219	0.550	0.149	7.666	< 0.001	(3.140, 5.299)
**Distance from the nearest medical and health service institution**						
< 1 km (reference group)						
1–3 km	−2.423	0.567	−0.074	−4.278	< 0.001	(−3.534, −1.313)
**Gender**	−1.573	0.467	−0.057	−3.369	0.001	(−2.489, −0.658)
**R**^**2**^	0.239					
**Model 2 (taking the two dimensions of trust as the independent variable)**						
Constant	22.930	1.193	–	19.225	< 0.001	(20.591, 25.269)
**Benevolence**	3.107	0.094	0.532	33.010	< 0.001	(2.922, 3.292)
**Insurance**						
Medical Insurance for Urban Employees (reference group)						
Medical Insurance for Urban Residents	6.804	0.856	0.132	7.945	< 0.001	(5.125, 8.483)
Basic Medical Insurance for Urban and Rural Residents	1.923	0.552	0.062	3.481	0.001	(0.840, 3.006)
**Region**						
Western province						
Central province	3.550	0.509	0.126	6.969	< 0.001	(2.551, 4.549)
**No. of visits in the past year**	−0.080	0.013	− 0.097	−6.053	< 0.001	(−0.106, − 0.054)
**Distance from the nearest medical and health service institutions**						
< 1 km (reference group)						
1–3 km	−2.351	0.529	−0.072	−4.443	< 0.001	(−3.389, −1.314)
**Gender**	−1.144	0.437	−0.042	−2.620	0.009	(−2.000, −0.288)
**R**^**2**^	0.336					

### Structural equation modeling

**Fig. 2:**
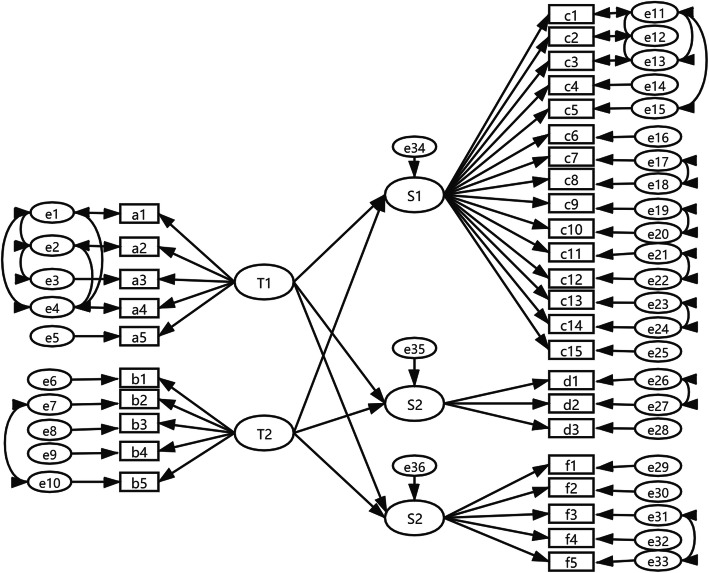
Structural equation model of trust in physicians and patient satisfaction

On the basis of our factor analysis, “T1 benevolence” and “T2 technical competence” were used as exogenous latent variables, while “S1 clinical behavior,” “S2 medical service continuity and cooperation,” and “S3 organization of care” were used as endogenous latent variables. The corresponding entries acted as observation variables to construct a structural equation model. The final structural equation model is depicted in Fig. 2, and the variables were showed in Table 7. For the fit indices, χ^2^/df, GFI, AGFI, NFI, IFI, and CFI were >  0.9, and the RMSEA was < 0.05, indicating good model fit (Table 8).

**Table 7:** The variables of the structural equation model

Variable	
**T1**	Benevolence
**a1-a5**	The items of “Benevolence” (eg. My doctor will do whateverit takes to provide me all the care I need.)
**T2**	Technical competence
**b1-b5**	The items of “Technical competence” (eg. My doctor’smedical skills are not as good as they should be.)
**S1**	Clinical behavior
**c1-c15**	The items of “Clinical behavior” (eg. Making you feelyou had time during consultations.)
**S2**	Continuity and cooperation
**d1-d3**	The items of “Continuity and cooperation” (eg. Knowingwhat s/he had done or told you during previous contacts.)
**S3**	Organization of care
**e1-e5**	The items of “Organization of care” (eg. Getting anappointment to suit you.)

**Table 8:** Fit indices of final model

Fit indices	GFI	AGFI	CFI	NFI	IFI	χ^2^/df	RMSEA
Reference value scale	> 0.9	> 0.9	> 0.9	> 0.9	> 0.9	< 5	< 0.08
Fitted value	0.929	0.915	0.942	0.933	0.942	7.322	0.049

^*a*^*GFI* Goodness of fit index; *AGFI* adjusted goodness of fit index; *CFI* comparative fit index; *NFI* normed fit index; *IFI* incremental fit index; *RMSEA* root mean square error of approximation

Results of the SEM indicated that benevolence positively influenced clinical behavior (0.940), organization of care (0.910), and continuity and cooperation (0.879), while technical competence negatively influenced clinical behavior (− 0.077), organization of care (− 0.136), and continuity and cooperation (− 0.064) (Table 9).

**Table 9:** Results of structural equation modeling

Path	Unstandardized regression weights	Standardized regression weights	*t*	Hypothesis (Y/N)
Benevolence→ Clinical behavior	1.607	0.940	25.493**	Y
Benevolence→ Continuity and cooperation	1.642	0.910	25.041**	Y
Benevolence→ Organization of care	1.716	0.879	26.800**	Y
Technical competence→ Clinical behavior	−0.169	−0.077	−4.116**	N
Technical competence→ Continuity and cooperation	−0.317	−0.136	−6.255**	N
Technical competence→ Organization of care	−0.161	−0.064	−3.001**	N

* < 0.05, ** < 0.01

## Discussion

In the present study, we found patients’ trust in their PCPs to be the strongest predictor of patient satisfaction. Among our sample population, both trust scores and satisfaction scores were relatively low. The median patient trust score was 2.4 per question, which is lower than the scores reported by Dong and Bao (3.1) [37], who used the same scale to conduct a trust survey of outpatients at Shanghai Third Grade Hospital. The score was also lower than scores reported in studies conducted overseas regarding patients’ trust in family physicians and primary care providers [30, 49]. A possible reason for this discrepancy is the gap between the service capacity of China’s primary hospitals in rural areas and patients’ demands regarding diagnosis and treatment, hindering the formation of a long-term stable partnership between doctors and patients [50]. In the present study, the median satisfaction score was 2.2 per question, far below the scores reported in studies of China’s urban community health service centers (over 4 per question, also measured using EUROPEP) [45, 51]; the scores in our study were also lower than those reported in a survey assessing patient satisfaction during GP visits across nine European countries [52]. A possible reason is that the present study targeted rural-based hypertension patients who, as a result of educational and social environmental factors, have poor self-care awareness and inadequately controlled high blood pressure [53]. This may result in an overreliance on doctors and higher numbers of PCP visits, thereby creating an impression that the physician is not sufficiently competent. Additionally, although China has formed a relatively comprehensive system for chronic disease prevention and treatment, there are still many issues in rural areas, such as the unreasonable allocation of medical and health resources, medical staff’s low enthusiasm, insufficient policies for hypertension prevention and treatment, and a lack of effective supervision and evaluation mechanisms for hypertension control [53]. Together, these problems have created a situation in which rural-based hypertension patients are less likely to have high satisfaction with PCPs, regardless of whether the issue is caused by the physician, other medical staff, or associated policies [28].

For the two dimensions of patient trust, the score for “technical competence” was higher than “benevolence,” suggesting that, in comparison to benevolence-associated aspects, when undergoing hypertension management in primary-health-care institutions, rural patients may pay more attention to physicians’ attitudes and communication skills, and may be more likely to feel that physicians are sufficiently clinically competent to address common medical problems, especially high blood pressure. The second linear regression, which set the two dimensions of trust as the independent variables, showed that the score for “benevolence” was significantly associated with an increase in satisfaction, while the score for “technical competence” had no impact. “Benevolence” represents physicians’ attitudes toward care and their communication competence [37]. Our findings indicate that patients’ perceptions regarding physicians’ levels of considerate communication are positively related to patient satisfaction [54]. A similar result was found in a study assessing factors that contribute to patients’ satisfaction with family physician consultations: most patients highlighted poor communication as a major factor that negatively affects the physician-patient relationship, rather than physicians’ professional competency [14].

The results of our multiple linear regression analysis and SEM differed slightly. In our SEM, all hypothesized paths were significant. Specifically, “benevolence” had a major positive impact on all three satisfaction dimensions. An expression of high trust in physicians’ “benevolence” indicates that patients believe that physicians care about them and are willing to devote notable time to their treatment [37]. Although patient satisfaction can be enhanced once a trusting relationship has been established, “technical competence” had a small but negative impact on all three satisfaction dimensions, directly contradicting our original hypothesis. This appears unusual but can be explained by the “customer-perceived quality of service theory” (similar to patient satisfaction, which can be understood as “patient-perceived quality of health service”). This theory holds that service quality comprises two parts: technical quality (the result of the service) and functional quality (the service process) [55]. Technical quality is a “hygiene factor” in regard to service quality, which means that high technical quality may not obviously improve patient-perceived health-service quality. Moreover, as a basic public health service, hypertension management is not very demanding for doctors in terms of technical competence; instead, there is a greater need for doctors to improve the service process and to make their patients feel their concern and that their communication is enjoyable [56]. In our study, although physicians’ technical competence may be similar, it was not the patients’ main concern. Instead, patients frequently made requests concerning the service process (or functional quality) provided by physicians, such as physician-based care, communication, and referral arrangements. Additionally, our investigation focused on physicians’ clinical behavior (including the patient-doctor relationship and communication, medical care, and information and support), organization of care, and continuity and cooperation, which mostly relate to the hypertension treatment service process. Thus, based on our results, patients are more likely to express their dissatisfaction with these dimensions when they have relatively high trust in their PCPs.

When examining the influence of socio-demographic and other variables, we found that patients who lived in the central province tended to have higher satisfaction than patients who lived in the eastern and western provinces. A study conducted in eight cities located in rural areas of China yielded similar results [53]. This region-based difference in satisfaction is potentially caused by differing health-service conditions and resident needs. For instance, patients in the eastern province may have had access to better health services, but their hypertension-related knowledge, particularly regarding risks and management, may have caused them to make more requests to visit their physicians. In the western province, financial limitations meant that the services patients received did not reach the standards of those of the eastern province; thus, patient satisfaction was lower. Notably, patients with more visits in the past year reported lower satisfaction levels. For hypertension patients, graded follow-up management is implemented based on blood pressure levels; more visits mean blood pressure is not being adequately controlled or indicate the presence of a more serious condition [6]. Such patients need medication, and their quality of life is more likely to be affected, thereby increasing the economic burden of the disease [55]. Patients who lived near health institutions were more satisfied owing to convenience and medical insurance also impacted patients’ satisfaction. For instance, patients insured with the Medical Insurance for Urban Residents (MIUR) and Basic Medical Insurance for Urban and Rural Residents were more satisfied. Compared with the NRCMS, MIUR reimburses for a wider range of drugs and has a relatively more convenient referral process [53, 57]. Basic Medical Insurance for Urban and Rural Residents is integrated into MIUR and NRCMS and features, within its scope, medical institutions and drugs that can be reimbursed under both types of insurance, thereby benefiting rural residents [58]. Thus, integrating medical insurance for urban and rural residents can improve patient satisfaction. Regarding gender, a consistent conclusion has not yet been established across the literature [14, 32]; however, our study showed that men had greater satisfaction with their family physicians. No other factors, including age, education, marital status, or self-reported status, had an influence in this regard, indicating that patient satisfaction is a universal phenomenon across these variables.

This study has several limitations. In particular, we did not measure the impact of physician-related characteristics and the regression models had a relatively small goodness of fit index (R^2^), indicating a limited ability to explain the variations in the dependent variables, but still within the acceptable range. However, we did not aim to predict patient satisfaction ratings; instead, we primarily aimed to identify the influence of patient trust and its dimensions on these ratings. Despite these shortcomings, by using SEM we have extended the current literature concerning relationships between trust and satisfaction among Chinese rural patients with hypertension, clarifying the influencing mechanism of the internal dimension.

## Conclusion

This study analyzed the effect of patient trust with their PCPS on satisfaction among rural-based patients with hypertension. Results indicated that during hypertension management, patients focus more on physicians’ benevolence than on their technical competence. Thus, medical humanities education should be emphasized for PCPs to improve the services they provide, as well as their service attitude. Concurrently, we found that physicians’ communication skills played an essential role in improving patients’ satisfaction. However, low overall trust and satisfaction among patients can negatively influence patients’ self-management and doctors’ enthusiasm, exerting a harmful influence on the effective management of hypertension. Thus, it is important for patients and physicians to establish a long-term, stable partnership.

Our findings regarding other factors, such as region- and health-insurance-based differences, indicate that the existing inequities between eastern, central, and western provinces as well as those between urban and rural areas must be addressed. Thus, future formulations of policies should fully consider regional characteristics and integrate medical insurance for urban and rural residents. To meet residents’ needs, services in eastern provinces should focus on improving efficiency, while services in western provinces need to improve in other areas, such as health facilities and the workforce.

## Data Availability

The datasets used during the current study are available from the corresponding author on reasonable request.

## Abbreviations

EUROPEP: European Task Force on Patient Evaluation of General Practice
GP: General Practitioner
KMO: Kaiser-Meyer-Olkin test
MIUR: Medical Insurance for Urban Residents
NRCMS: New Rural Cooperative Medical Scheme
PCP: Primary Care Physician
SEM: Structural Equation Model
VIF: Variance Inflation Factor
WFPTS-C: Chinese version of the Wake Forest Physician Trust Scale
